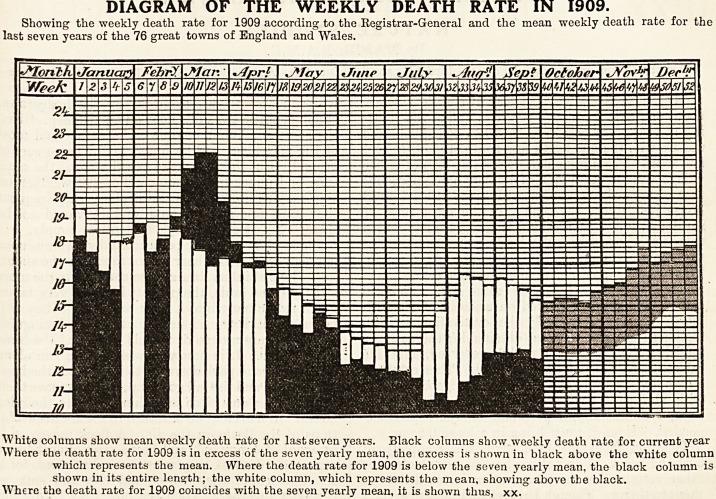# Diagram of the Weekly Death Rate in 1909

**Published:** 1909-11-20

**Authors:** 


### Public Health and Hygiene.

### DIAGRAM OF THE WEEKLY DEATH RATE IN 1909.

Showing the weekly death rate for 1909 according to the Registrar-General and the mean weekly death rate for the last seven years of the 76 great towns of England and Wales.

White columns show mean weekly death rate for last seven years. Black columns show weekly death rate for current year
Where the death rate for 1909 is in excess of the seven yearly mean, the excess is shown in black above the white column which represents the mean. Where the death rate for 1909 is below the seven yearly mean, the black column is shown in its entire length; the white column, which represents the mean, showing above the black.
Where the death rate for 1909 coincides with the seven yearly mean, it is shown thus, xx.

The diagram which we publish above tells its own story so clearly as to leave no occasion for description. It exhibits, however, such remarkable features in the combined death-rates of the 76 great towns as at once to arrest attention. The mean weekly death-rate of the last seven years, although in some measure it typifies the seasonal variations in mortality, is itself component of such violent deviations from the normal curve which would be represented by the mean of a larger series, that the peculiar features of the current year are masked rather than exaggerated.

We have had a succession of abnormally wet and cold summers and of mild winters, all of which have materially disturbed the normal course of seasonal mortality. A hot, dry summer is associated with a high mortality among infants, chiefly from zymotic diarrhœa; a rigorous winter is inimical to life in the aged and infirm, and the mortality bills of severe winters are swelled by deaths from pulmonary affections.

Accustomed as we are in these islands to departures from uniformity in the matter of the weather, it has to be confessed that its vagaries in recent years have left scope for astonishment even in a people so inured as our own. The effect of such climatic variations upon crops is of vulgar interest and observation, but the influence of this circumstance upon seasonal mortality has singularly escaped the common notice.

It is true we have our proverb that "a green yule makes a fat kirkyard"; but however, if at all, true this may have been in the altered conditions of the past, the opposite holds to-day. Mild winters and cold summers mean a low mortality for our urban population, and this chiefly due to an increased survival of persons at the extremes of life.

It is tempting to speculate on the influence which these results may have upon the birth-rate, which if they were continuously operative would necessarily decline both as a direct and indirect effect of these changes. They modify the age constitution of the population, and thus directly affect the birth-rate; they conserve infant life and thus diminish what we may describe as the natural demand for children; and there is increasing evidence to show that this is a factor in the problem of the declining birth-rate.

It would, however, be impossible to disentangle the sequences which flow from these passing changes in the vital phenomena of the time, and we must content ourselves with noting the facts.

Unless the meteorological changes which have characterised recent years have come to stay, they will have left an impress which will distinguish the period by a mortality curve of unique character. In this connection, however, it is to be noted that the striking peak in March, shown in the diagram in black, has presumably little to do with the prevailing weather, since it was due to an epidemic of influenza. It is because crude mortality curves are subject to disturbance from accidents of this kind that their meaning is so difficult to interpret.

## Figures and Tables

**Figure f1:**